# Retrograde intrarenal surgery in a non-fused left crossed ectopic kidney with large renal calculus: a case report and review of literature

**DOI:** 10.1093/omcr/omag162

**Published:** 2026-09-02

**Authors:** Adeel Anwaar, Inam Ul Haq, Abdul Munim Khan, Farooq Hameed, Aymar Akilimali

**Affiliations:** Pakistan Kidney and Liver Institute and Research Center, Lahore, 54920, Punjab, Pakistan; Rashid Latif Medical College, Lahore, 54920, Punjab, Pakistan; Milat Hospital, Sadiqabad, 64530, Punjab, Pakistan; Urology Suite, Midcity Hospital, Lahore, 54920, Punjab, Pakistan; Department of Research, Medical Research Circle (MedReC), Goma, Democratic Republic of Congo

**Keywords:** crossed renal ectopia (non-fused), retrograde intrarenal surgery (RIRS), renal calculi, anomalous kidney anatomy, flexible ureteroscopy

## Abstract

Crossed renal ectopia is a rare congenital anomaly, with non-fused variants being exceptionally uncommon. The management of nephrolithiasis in such kidneys is challenging due to the abnormal anatomy, malrotation, and altered ureteral course. We report a case of a non-fused left crossed ectopic kidney with a 21 × 14.5 mm with density of 1250 HU renal calculus successfully managed with retrograde intrarenal surgery (RIRS), achieving complete stone clearance (SC). This case highlights the feasibility of RIRS, even in complex renal anomalies with a significant stone burden. A review of the existing literature further supports the expanding role of RIRS in anomalous kidneys.

## Introduction

Crossed renal ectopia (CRE) is a rare congenital anomaly in which one kidney crosses the midline and is located contralateral to the other kidney. In the majority of cases, the ectopic kidney is fused with the orthotopic kidney, accounting for nearly 90% of presentations, whereas the non-fused variant remains exceptionally rare and is sparsely reported in the literature. The overall incidence of CRE ranges from 1 in 1000 to 1 in 7500 autopsies, with many cases remaining clinically silent and detected incidentally during imaging for unrelated conditions [1, 2].

Despite being asymptomatic in many individuals, crossed ectopic kidneys are predisposed to complications such as nephrolithiasis, urinary tract infection, and obstruction. These complications primarily arise due to abnormal renal rotation, impaired drainage, and anomalous vascular supply. The management of renal calculi in such anomalous kidneys is particularly challenging because of the distorted anatomy, atypical calyceal orientation, and ureter crossing the midline before entering the bladder, which complicates both access and navigation [2].

Traditionally, percutaneous nephrolithotomy (PCNL) is the preferred treatment for large renal stones in anomalous kidneys. In anomalous configurations, some literature suggests that SWL outcomes can be optimized by using contralateral coupling of the SWL cushion to achieve appropriate targeting. However, SWL is bypassed or declined in high density stones. Achieving safe percutaneous access in ectopic kidneys is often difficult because of the presence of interposed bowel loops, altered anatomical landmarks, and unpredictable vascular variations, which increase the risk of complications. With advancements in endourology, retrograde intrarenal surgery (RIRS) has emerged as a minimally invasive alternative, offering the advantage of a natural orifice approach with reduced morbidity. Recent studies have demonstrated the favourable outcomes of RIRS in anomalous kidneys, including higher stone-free and lower retreatment rates compared to those of shock wave lithotripsy [3]. However, reports of RIRS in non-fused crossed ectopic kidneys, particularly in the presence of a large stone burden, remain extremely limited.

## Case report

A 26-year-old male presented with a 2-year history of mild-to-moderate right flank pain. There was no history of hematuria, fever, dysuria, pyuria, urinary retention, or prior stone passage in the patient. The patient denied nausea, vomiting, weight loss, trauma, or anticoagulant use.

The patient had no significant medical or surgical history. The patient reported the intake of herbal medications. His personal and family histories were unremarkable.

**Figure 1:**
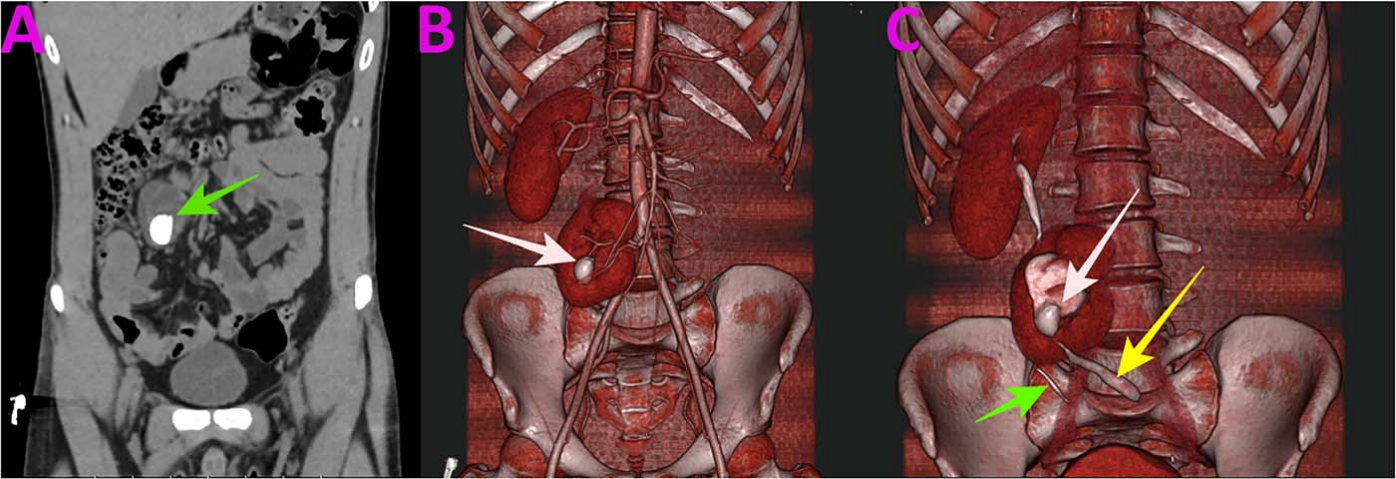
Left kidney measures 8.2 cm in bipolar dimension and is seen ectopically located in right hemi-abdomen at L3-L4 level, close to the midline. A calculus measuring 21 × 14.5 mm with density of 1250 HU is seen at PUJ, resulting in upstream mild hydronephrosis. (green arrow in A figure, white arrow in figure B and C) left ureter shows orthotopic insertion at left VUJ (yellow arrow in figure C).

On examination, the patient was well-oriented, with stable vital signs. There was no evidence of pallor, jaundice, lymphadenopathy, or edema. Abdominal examination revealed a soft, non-tender abdomen with non-palpable kidneys and bladder. Laboratory investigations were within normal limits. Ultrasonography of the kidneys, ureters, and bladder (KUB) demonstrated a non-fused left crossed ectopic kidney located on the right side, harboring a renal calculus measuring 21 × 14.5 mm. Contrast-enhanced and non-contrast CT scans confirmed a 21 × 14.5 mm renal stone with a density of 1250 HU seen at proximal ureteric junction (PUJ), resulting in upstream mild hydronephrosis, and revealed the complex renal anatomy associated with the crossed ectopia (Fig. 1). The ureter crossed the midline and inserted normally into the urinary bladder.

Management options, including extracorporeal shock wave lithotripsy (ESWL), percutaneous nephrolithotomy (PCNL), and retrograde intrarenal surgery (RIRS), were discussed with the patient. Given the relatively high stone density (1250 HU), the likelihood of successful stone clearance with ESWL was considered low. Considering the anatomical complexity, significant stone burden, and the anticipated challenges associated with percutaneous access, both PCNL and RIRS were carefully evaluated. RIRS was ultimately selected as the preferred minimally invasive treatment option, and the patient consented to proceed with this approach. The patient was counseled regarding the possibility of converting to a laparoscopically assisted puncture and PCNL if RIRS could not be successfully performed.

**Figure 2:**
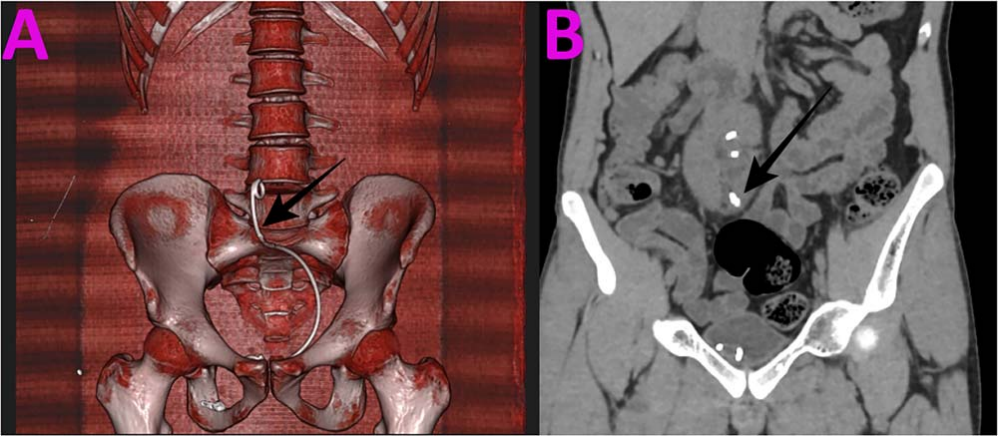
no residual stone after procedure, black arrow shows stent in situ.

The procedure was performed under general anesthesia (GA), which was selected due to the anticipated long duration, complex anatomy, and the need for optimal respiratory control and patient comfort. Cystoscopy was performed, revealing a left ureteral orifice with orthotopic insertion. Retrograde pyelography was subsequently performed to evaluate the upper tract anatomy. A 0.035 Inch glidewire was advanced into the left ureter that crossed the midline. A 35 cm, 9/11 Fr Hugemed ureteral access sheath was inserted smoothly over glide wire. A 7.5 Fr Hugemed® flexible ureteroscope was advanced in access sheath that revealed altered calyceal anatomy, but successful navigation. Laser lithotripsy was performed with a 100-W Quanta® Holmium laser using a 200-μm fiber. Stone dusting was executed via magneto-modulation settings (0.5 J, 20 Hz), followed by pop-corning (1.2 J, 15 Hz) to clear residual larger fragments. A small stone was retrieved using dormia basket for stone analysis. Complete clearance was confirmed by endoscopy. A 6/22 Fr/cm double-J ureteral stent was placed due to the unavailability of a shorter-length stent. The total lasing duration was 52 minutes, with an overall operative time of 87 minutes. The postoperative course was uneventful. Follow-up was performed using CT KUB, obtained at 3 weeks interval (Fig. 2). The double-J stent was removed 3 weeks after surgery. Postoperative stone composition analysis confirmed calcium oxalate monohydrate. Although a metabolic workup was recommended to assess for underlying metabolic risk factors, the patient was lost to follow-up.

## Discussion

The management of nephrolithiasis in crossed renal ectopia remains challenging because of the associated anatomical abnormalities, including ectopic renal location, malrotation, aberrant vascular supply, and altered pelvicalyceal orientation. These factors can significantly complicate surgical access and stone clearance. The challenges are particularly pronounced in non-fused crossed renal ectopia, a rarer variant in which the kidneys remain separate, resulting in unpredictable collecting system anatomy and ureteral course. Traditionally, percutaneous nephrolithotomy (PCNL) has been considered the preferred treatment for large renal calculi in anomalous kidneys. However, the procedure may be technically demanding and carries a higher risk of injury to adjacent organs and anomalous vessels because of distorted anatomical relationships [4]. While ESWL has been reported as a treatment option in previous cases of crossed renal ectopia, the high stone density in our patient made this approach less favorable because of the anticipated lower fragmentation success.

Recent advances in endourological technology have expanded the role of retrograde intrarenal surgery (RIRS) in the management of stones within anatomically abnormal kidneys. Improvements in flexible ureteroscope design, enhanced deflection capabilities, and advances in laser lithotripsy have enabled successful navigation and treatment in complex renal anatomies. Several studies have demonstrated favourable outcomes with RIRS in anomalous kidneys, reporting high stone-free rates and low complication rates. Comparative analyses have also shown superior stone clearance and lower retreatment rates with RIRS compared with shock wave lithotripsy in this patient population [3]. Furthermore, successful use of RIRS has been reported in pelvic kidneys and cross-fused renal ectopia, supporting its growing role as a minimally invasive treatment option for renal calculi in anomalous kidneys (Table 1) [5–8].

**Table 1:** Summary of previously reported RIRS cases in ectopic kidneys.

Author	Year	Type of anomaly	No of cases	Stone size	Procedure	Outcome
Resorlu *et al*. [5]	2015	Cross-fused ectopic kidney	1	Not specified	RIRS	Successful
Krishnaprasad *et al*. [6]	2022	Pelvic ectopic kidney	1	1 cm	RIRS	Complete clearance
Reggio *et al*. [8]	2022	Pelvic ectopic kidney	1	12 mm	RIRS	Complication (bleeding)
Present case	2026	Non-fused crossed ectopic kidney	1	21 mm	RIRS	Complete clearance

Despite these encouraging results, RIRS in crossed ectopic kidneys remains technically demanding. The ureter often traverses the midline, creating acute angulations that may hinder ureteroscopic advancement and complicate ureteral access sheath placement. In addition, altered calyceal orientation and limited working space can reduce maneuverability and make complete stone clearance challenging. Ssuccessful outcomes depend on detailed preoperative imaging, meticulous surgical planning, appropriate instrument selection, and considerable endourological expertise. In the present case, these anatomical challenges were successfully managed through careful preoperative assessment and the use of modern flexible endourological equipment, resulting in complete stone clearance without complications.

The present case is notable for two reasons. First, it involves a non-fused crossed renal ectopia, an exceptionally rare congenital anomaly for which published experience remains limited. Second, the patient had a relatively large renal stone burden of 21 mm. According to conventional treatment paradigms, stones of this size would frequently favor a percutaneous approach. Stone clearance was achieved using RIRS alone, demonstrating that the indications for RIRS may extend beyond traditional limits in selected patients when performed by experienced surgeons.

This case adds to the growing evidence supporting RIRS as a primary treatment option for renal stones in patients with complex renal anomalies. The procedure offers several advantages, including avoidance of percutaneous access-related morbidity, reduced postoperative discomfort, shorter hospital stays, and faster recovery. As endourological technology and surgical expertise continue to evolve.

This case demonstrates the feasibility of RIRS in a carefully selected patient with non-fused crossed renal ectopia and a substantial stone burden. While larger studies are required to establish definitive treatment recommendations, the present report highlights RIRS as a valuable minimally invasive alternative to percutaneous surgery in this rare anatomical setting.
